# Diagnosis of Drowning and the Value of the Diatom Test in Veterinary Forensic Pathology

**DOI:** 10.3389/fvets.2019.00404

**Published:** 2019-11-14

**Authors:** Giuseppe Piegari, Davide De Biase, Ilaria d'Aquino, Francesco Prisco, Rosario Fico, Raffaele Ilsami, Nicola Pozzato, Angelo Genovese, Orlando Paciello

**Affiliations:** ^1^Unit of Pathology, Department of Veterinary Medicine and Animal Production, University of Naples Federico II, Naples, Italy; ^2^National Center for the Forensic Veterinary Medicine, Istituto Zooprofilattico Sperimentale delle Regioni Lazio e Toscana, Grosseto, Italy; ^3^Verona Diagnostic Laboratory, Istituto Zooprofilattivo Sperimentale delle Venezie, Legnaro, Italy; ^4^Department of Biology, University of Naples Federico II, Naples, Italy

**Keywords:** veterinary forensic pathology, forensic medicine, drowning, diatoms, diatoms test

## Abstract

The detection of diatoms into the organs is considered an important “biological marker” for the diagnosis of drowning in human pathology, but it still has a high possibility for false positive results. The aims of this study were: (1) to evaluate the contribution of pathological examination in drowning cases and (2) to investigate the differences in the number and location of diatoms between animals who died in drowning and non-drowning conditions. For these purposes, 30 dead adult dogs were selected for the study and subdivided into five groups. The group A comprised six cadavers dead for drowning; the group B comprised six control animals; the groups C, D, and E comprised six animals dead for causes other than drowning and subsequently immersed in water for 24, 48, and 72 h, respectively. On each animal, a complete macroscopic and histological examination and diatom test were performed. Diatoms test and quantification were also performed on drowning mediums. Pathological findings of the animals in the group A showed pulmonary congestion, oedema, and hemorrages in the lung. However, similar injuries were also observed in control and experimentally submerged cadavers. In contrast, we observed a statistically differences between drowning animals and all experimentally submerged groups and control animals regarding diatom numbers recovered from organ tissue samples (*p* < 0.05). Therefore, these findings suggest that the number of diatoms may be used as a valid tool to differentiate animals who died in drowning and non-drowning conditions, even if the latter were found in an aquatic environment.

## Introduction

The World Health Organization (WHO) defines drowning as a “process of experiencing respiratory impairment from submersion/immersion in liquid” (1). The diagnosis of drowning is reported in the literature as one of the most difficult in the field of human forensic pathology (2, 3). Although drowning mechanisms and the associated injuries have been described extensively in the human medical literature, this diagnosis remains a difficult one to confirm (3). Indeed, a wide range of information is required to issue a diagnosis of drowning in human forensic pathology, such as information about the clinical history of the victim, crime scene analysis, testimony and physical evidence, microscopic and histological findings, and ancillary test results (3). In drowned bodies, the most common lesions reported in the literature are pulmonary congestion, pulmonary oedema and pulmonary hemorrhage, foam in the trachea, mouth and nasal cavity, right ventricular distention and drowning medium into the stomach (3–6). Similarly, the main histological injuries include intra-alveolar hemorrhage, pale or proteinaceous fluid within alveoli, alveolar overdistension, aspiration of gastric fluids, and foreign material in alveolar space (3). Over the last years, many ancillary tests have been proposed for the diagnosis of drowning in human forensic pathology, such as microbiological and electrolyte tests, and real-time PCR assays (3, 7–10). However, among these, no one has obtained as much attention as the diatom test. The detection of diatoms into the organs is currently considered an important “biological marker” for the diagnosis of drowning in human forensic pathology (10, 11). Indeed, the diatoms test is based on the principle that diatoms can be detected in the organs of drowned victims if they aspirated diatom-rich water into the lung before death (10). Diatoms are unicellular, photosynthetic and autotrophic organisms that live both in fresh water and saltwater. Usually, the size range of diatoms (between 20 and 200 μm) and their morphology allow them to percolate through the alveolo-capillary barrier and subsequently enter into the bloodstream during the pre-agonic state of drowning (8, 10, 12). Although the diatom test is a valid test to support the diagnosis of drowning, its sensitivity and specificity is still controversial; the main limitation is based on the potential post-mortem contamination of the organs due to the ubiquitous distribution of diatoms. Furthermore, some authors have suggested the possibility of false positive diatoms results due to ante-mortem penetration of diatoms in the bloodstream through the intestinal or respiratory tract (13, 14). Finally, negative diatom results have been reported in tissues of humans who died in drowning condition (15). In veterinary forensic pathology, the lack of medical literature, associated with the wide range of animals of interest, makes the diagnosis of drowning even more difficult than a drowning diagnosis for humans. In addition, although the use of the diatom test has been sporadically reported in veterinary medicine, to the best of the authors' knowledge, no study has evaluated its sensitivity and specificity in veterinary contest (10). Finally, only a few reports have investigated macroscopic and histological injuries in cases of accidental and nonaccidental drowning in animals of veterinary forensic interest (3). In light of these observations, the aims of this study are as follows: (1) to evaluate the macroscopic and microscopic findings in drowned animals and, specifically, to evaluate the contribution of necropsy and histological examination to determine the cause of death in drowning cases in veterinary forensic pathology; (2) to investigate the differences in the number and location of diatoms between animals who died in drowning and non-drowning conditions; and (3) to investigate the correlation between the time of permanence in water and the number and location of diatoms in animals dead for causes other than drowning and subsequently used for experimental submersions in standard conditions.

## Materials and Methods

### Study Design

Thirty dead adult dogs [medium-sized animals, age range 6–12 years, mean age 9.33 (SD 2.38)], were enrolled for the study and divided into five groups. Group A comprised six cadavers dead from drowning and recovered from aquatic environments (time since death <48 h); the group B comprised six control animals dead for causes other than drowning; the group C comprised six animals dead for causes other than drowning and subsequently immersed for 24 h in water; the group D comprised animals dead for causes other than drowning and subsequently immersed for 48 h in water. Finally, the group E comprised animals dead for causes other than drowning and subsequently immersed for 72 h in water. All post-mortem experimental submersions of the cadavers in groups B, C, and D were performed at room temperature (22°C) using water with a range of diatoms of 754–785 (769 ± 15 SD) per 100-μl of pellet obtained from 10 ml of sample.

### Water Samples

Between 13 January, 2018 and 20 February, 2019, samples of water were obtained from the five sites where the drowned animals (group A) were recovered: a well and pond located in the countryside of the Veneto region, where the case #1, case #2, case #3 were recovered, the Brulamacca canal located in the Toscana Region where the case #4 was recovered and the Mediterranean Sea in Agropoli and the Mediterranean Sea in Naples, where the case #5 and case #6 were recovered (**Table 3**). The samples were collected using plastic sterile containers. the samples were used for diatoms identification and counting of the five water sites.

### Macroscopic and Histological Examination

Post-mortem examination was performed in all cadavers. The forensic necropsies were performed in the necropsy room of the Department of Veterinary Medicine and Animal Production of the University of Naples “Federico II” with a standard forensic necropsy protocol previously described by Piegari et al. (16). In addition, lung samples were collected for histopathologic examination; samples were fixed in 10% neutral buffered formalin, embedded in paraffin, sectioned at 4 microns and stained with hematoxylin and eosin (HE) for morphological evaluation of lesions (17). For the purposes of this study, multiple samples were taken from left cranial and caudal lobes and right cranial, middle, accessory and caudal lobes, in order to obtain a representative evaluation of the whole organ.

### Diatom Test

According to the protocol previously described by Fucci et al. (10), for control, experimentally submerged cadavers and drowned animals, five grams of lung, liver, kidney, brain, spleen, and bone morrow, were processed for diatom test performed with the hydrogen peroxide digestion method. Finally, the same aforementioned protocol was used to process 10 ml of each drowning medium of the animals in group A. For the purposes of this study, we used the hydrogen peroxide method to better preserve the diatoms during the extraction process. Furthermore, to avoid contamination, an internal protocol was used to take the samples and to process them in the laboratory. This internal protocol included the use of sterile instruments for each sample and the use of Diatom-free water for washing. Table 1 summarizes the diatom test steps and the protocol used to avoid diatom contamination.

**Table 1:** Diatom test protocol and internal protocol used to avoid diatom contamination.

**Diatom test step**	**Internal protocol to avoid diatom contamination**
Necropsy	Sterile blades, change of the blade after skinning to avoid skin contact with organs
Sample collection	Sterile blade, separate instruments for each sample, diatom-free glass containers
Samples treated with HCL (20%) for 24 h	Sterile blade, separate instruments for each sample, diatom-free glass containers
Washing	Diatom-free water for washing
Samples treated with H_2_O_2_ (40%)	diatom-free glass containers
Overnight sedimentation and decanting of excess liquid	//
Sediment centrifuged three times at 1,000 rpm and washed	Diatom-free water for washing, diatom-free glass containers

### Diatom Quantification and Statistical Analysis

After hydrogen peroxide digestion method and centrifugation of the samples, 100-μl of pellet was transferred onto microscope slides and mounted with Naphrax resin (Brunel Microscopes Ltd., Chippenham, UK). Diatom slides were evaluated using a standard light microscope at 40× magnification for diatom counting and 100× for diatoms identification. Diatoms were evaluated and classified according to the international literature (18–20). The SPSS 20.0 package (SPSS Inc., Chicago, IL, USA) was used for statistical analysis of the data. The Mann–Whitney test, a nonparametric test for two independent samples, was used to assess the differences in diatom number between groups. *P*-values < 0.05 were considered statistically significant.

## Results

### Forensic Necropsy

In all animals in group A, we observed mild to moderate pulmonary congestion and moderate to severe pulmonary oedema. Furthermore, in 1 case, we found water in the stomach and in 3 out of 6 cases, pulmonary hemorrhages and right ventricular distention. In addition, in 4 out of 6 cases, we observed moderate to severe pulmonary emphysema. Finally, in 1 case, fracture of the ribs and subcutis hemorrhages were also observed. However, similar lesions were found in control and experimentally submerged cases (Table 2).

**Table 2:** Macroscopic lesions of drowning observed in study animals stratified by group.

**Group**	**Wet hair coat**	**Pulmonary congestion**	**Pulmonary edema**	**Pulmonary hemorrhages**	**Pulmonary emphysema**	**Right ventricular distension**	**Foreign material in stomach**	**Traumatic lesions**	**Cause of death**
GA.1^*^	X	X	X	X	X	–	X	–	Drowning
GA.2	X	X	X	X	–	X	–	–	Drowning
GA.3	X	X	X	X	X	–	–	X	Drowning
GA.4	X	X	X	–	–	X	–	–	Drowning
GA.5	X	X	X	–	X	–	–	–	Drowning
GA.6	X	X	X	–	X	X	X	–	Drowning
GB.1	–	X	X	–	–	–	–	X	Blunt trauma
GB.2	–	X	X	–	–	–	–	–	Euthanasia
GB.3	–	X	X	–	X	X	–	–	Dilatative cardiomyopathy
GB.4	X	X	X	X	X	–	–	X	Blunt trauma
GB.5	X	X	X	X	–	–	–	X	Blunt trauma
GB.6	X	X	X	X	X	X	–	X	Blunt trauma
GC.1	X	X	X	–	–	–	–	–	Euthanasia
GC.2	X	X	X	X	X	X	–	X	Blunt trauma
GC.3	X	X	X	–	–	X	–	–	Dilatative cardiomyopathy
GC.4	X	X	X	X	X	X	–	–	Kidney failure
GC.5	X	X	–	–	–	–	–	X	Traumatic Brain Injury
GC.6	X	X	X	–	–	X	–	–	Hypovolemic shock
GD.1	X	X	X	–	–	X	–	–	Euthanasia
GD.2	X	X	X	X	X	X	–	–	Dilatative cardiomyopathy
GD.3	X	X	X	–	X	X	–	–	Splenic cancer
GD.4	X	X	X	–	–	X	–	–	Euthanasia
GD.5	X	X	X	X	X	–	–	–	Septic shock
GD.6	X	X	X	–	–	X	–	–	Euthanasia
GE.1	X	X	X	X	X	X	–	–	Euthanasia
GE.2	X	X	X	–	X	X	–	–	Euthanasia
GE.3	X	X	X	–	X	X	–	–	Euthanasia
GE.4	X	X	X	X	X	X	–	–	Septic shock
GE.5	X	X	X	–	X	X	–	–	Euthanasia
GE.6	X	–	–	–	–	–	–	X	Traumatic brain injury

* *Group A (GA), Group B (GB), Group C (GC), Group D (GD), Group E (GE)*.

### Histological Analysis

**Figure 1:**
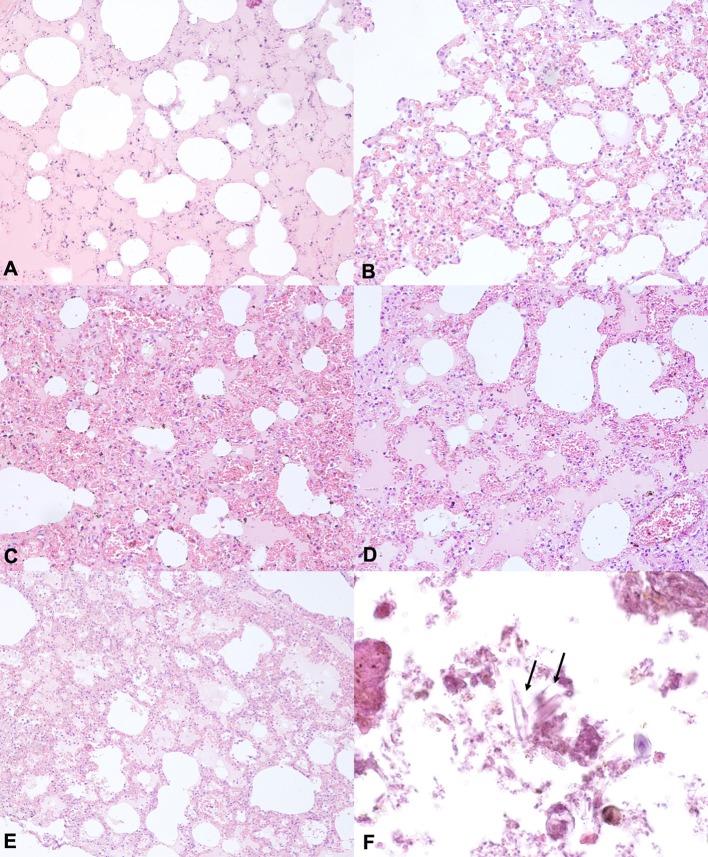
Representative H&E –stained sections from lung tissues of animals of the groups **(A–C)**. **(A)** Lung section from drowned animals showing proteinaceous fluid within alveoli. **(B)** Lung section from downed animals showing mild to moderate vasodilatation and emphysema. **(C,D)** Lung section from animals of the group B showing proteinaceous fluid in alveolar space, vasodilatation and emphysema. **(E)** Lung section from animals of the Group C showing proteinaceous fluid within alveoli, vasodilatation and intra-alveolar hemorrhages. Hematoxylin and eosin stain, original magnification 20×. **(F)** Lung section from animals in the group A showing foreign and unstained material, characterized by small and elongate structures, often with multiple striae perpendicular to their major axis, consisting of diatoms Hematoxylin and eosin Stain, original magnification 10×.

The histological examination performed on lung samples of the animals in group A revealed, in all assessed cases, moderate to severe pale or proteinaceous fluid within alveoli. In addition, in 3 out 6 cases, we observed multifocal intra-alveolar hemorrhages, mild to moderate vasodilatation and rupture of several alveolar walls. Moreover, a mild interalveolar infiltrate of macrophages was also observed in 1 out of 6 cases. However, similar injuries were observed in the lungs of animals in control and experimentally submerged groups. Indeed, in all animals in group B, we found mild to moderate pulmonary oedema and vascular congestion. In 3 out of 6 cases, we also observed multifocal hemorrhages and a mild infiltration of neutrophil granulocytes and macrophages. Similarly, in all animals in group C, we observed moderate to severe intra-alveolar oedema associated with vascular congestion and, in 2 out of 6 cases, intra-alveolar hemorrhages. A mild to moderate pulmonary oedema was also observed in Groups D and E. Finally, as regard the animals in group A, in only one case, the histopathological examination showed foreign material in alveolar spaces, which, by morphology, could be compatible with diatoms. In particular, the histopathological examination allowed the detection of foreign and unstained material, characterized by small and elongate structures, often with multiple striae perpendicular to their major axis. In contrast, in control and experimentally submerged cases, the histopathological examination did not allow us the detection of foreign and unstained material, compatible with diatoms or other algae (Figure 1).

### Diatoms in Drowning Medium

The ranges of diatom counts per 100 μl of pellet obtained from 10 ml of water samples of Mediterranean sea in Naples and Agropoli, well and pond located in the countryside region of Venezia and the Brulamacca canal located in the Toscana Region, were 310–383 (342 ± 37), 452–461 (458 ± 4), 720–782 (749 ± 31), 2,323–2,850 (2,524 ± 284), and 560–581 (571 ± 1,069) frustules, respectively (Table 3). The most common diatoms detected in our study belonged to the genus *Nitzschia, Navicula, Cymbella, Cyclotella, Fragilaria, Gomphonema, Amphora, Bacillaria, Cocconeis*, and *Craticula*.

**Table 3:** No. of diatoms per 100 μl recovered from drowning mediums and animal tissue samples.

**Animals**	**Site of recovery**	**Diatoms in water samples**	**Lung**	**Liver**	**Kidney**	**Brain**	**Spleen**	**Bone morrow**
GA.1^*^	Well	720–782 (749 ± 31)	29	6	2	n/d^**^	n/d	n/d
GA.2	Well	720–782 (749 ± 31)	28	5	2	n/d	n/d	n/d
GA.3	Pond	2.323–2850 (2.524 ± 284)	91	7	2	0	6	3
GA.4	Brulamacca canal	560–581 (571 ± 10)	37	2	3	0	2	2
GA.5	Mediterranean Sea in Naples	310–383 (342 ± 37)	29	4	2	0	3	0
GA.6	Mediterranean Sea in Agropoli	452–461 (458 ± 4)	27	2	1	0	3	0
GB.1	Terrestrial environment	–	0	0	0	0	0	0
GB.2	Terrestrial environment	–	0	1	0	0	2	0
GB.3	Terrestrial environment	–	0	0	0	0	0	0
GB.4	Terrestrial environment	–	0	0	0	0	0	0
GB.5	Terrestrial environment	–	0	0	0	0	0	0
GB.6	Terrestrial environment	–	0	0	0	0	0	0

* Group A (GA), Group B (GB);

** *no data*.

### Diatoms in Drowned and Control Animals

**Figure 2:**
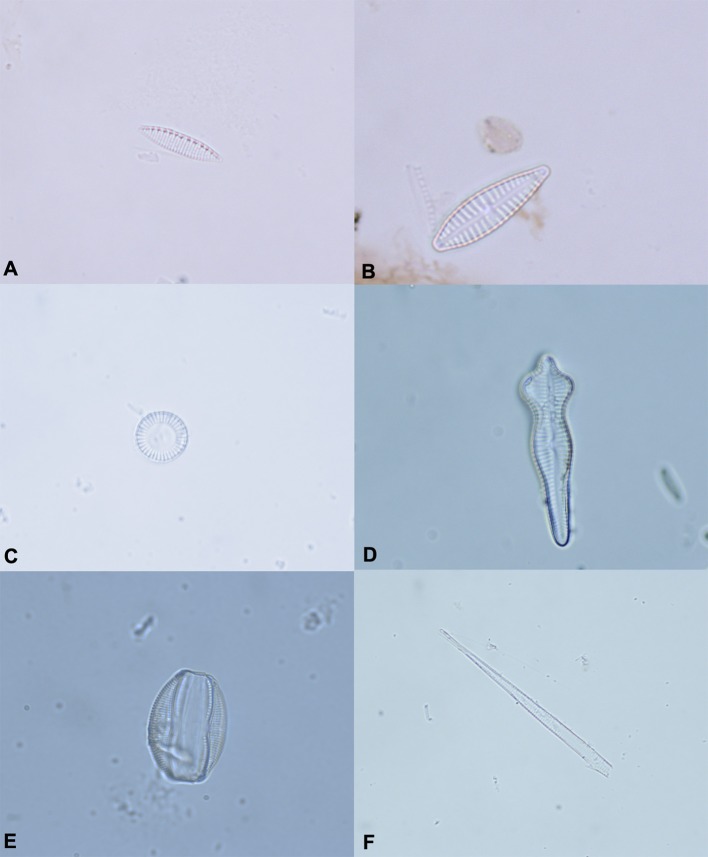
The most common diatoms identified in animals of the Group A**. (A)** *Nitzschia* spp. **(B)** *Gomphonema micropus*. **(C)** *Cyclotella* spp. **(D)** *Gomphonema acuminatum*. **(E)** *Amphora ovalis*. **(F)** Fragment of diatom. Original magnification 100×.

The diatoms test allowed us to observe a different diatom diffusion between drowned and control animals. In particular, diatoms were detected in the lung, liver and kidney of all animals in group A. Furthermore, diatoms were observed in the spleen of 3 out of 6 cases and in bone morrow tissues of 2 out of 6 animals. With regard to the animals in group B (control animal), no diatoms were detected in 5 out of 6 cases, while in one case, diatoms were recovered from spleen and liver tissue samples. The most common diatoms detected in the animals in group A belong to the genus *Nitzschia (Nitzschia capitellata Hustedt in Schmidt, Nitzschia dissipata, Nitzschia palea (Kützing) Smith, Nitzschia microcephala Grunow in Cleve & Möller, Nitzschia recta Hantzsch in Rabenhorst, Nitzschia subtilis*)*, Navicula, Cymbella, Cyclotella (Cyclotella meneghiniana Kutzing, Cyclotella ocellata Pantocsek), Fragilaria (Fragilaria construens f. binodis (Ehrenberg) Hustedt*) *Gomphonema (Gomphonema acuminatum, Gomphonema micropus (Kützing), Gomphonema parvulum (Kützing) Kützing*), Amphora (*Amphora inariensis Krammer, Amphora ovalis (Kutzing) Kutzing*), *Bacillaria (Bacillaria paxillifera Hendey)*, and *Cocconeis (Cocconeis placentula Ehrenberg)* and matched with the respective drowning mediums. Furthermore, all diatoms recovered from control animal tissue samples belonged to the genus *Navicula* (Figure 2).

In addition, we observed differences in diatom number between drowned and control animals. Overall, the number of diatoms in each assessed organ of the drowned animals ranged from 0 to 91 frustules/100 μl, while those in control animals ranged from 0 to 2. In addition, among drowned animals, we observed higher diatom numbers in lung tissue samples than those detected in other organs. In contrast, all the diatoms detected in control animals were in organs other than lungs.

Among drowned cases, the highest diatoms number was observed into the organs of the dog recovered in the pond located in the countryside region of Venezia. In this case, the range of diatoms density of the drowning medium was calculated as 2,323–2,850 (2,524 ± 284 SD)/100 μl of pellet and contained the highest levels of diatoms among the assessed drowning mediums (Table 3).

Finally, the Mann-Whitney test showed differences in diatom numbers between the drowned (Group A) and control group (Group B) (Table 4). In particular, we observed significantly lower diatom numbers in the lung, liver, kidney, and spleen of the control group (Group B) compared to the drowned animals (Group A).

**Table 4:** Mann–Whitney rank sum test between control and drowned animals.

**Animals**	**Lung**	**Liver**	**Kidney**	**Brain**	**Spleen**	**Bone morrow**
Median (group A)	29	4	2	0	3	1
Median (group B)	0	0	0	0	0	0
*P*-value	<0.05	<0.05	<0.05	>0.05	<0.05	>0.05

### Post-mortem Diatom Penetration

Diatoms were detected in the lungs of all experimentally submerged cadavers (group C = 24 h in water; group D = 48 h in water; and group E = 72 h in water). In addition, diatoms were recovered from liver and spleen tissue samples of 1 animal in group E (72 h in water). Furthermore, The most common diatoms detected in experimentally submerged cadavers belong to the genus *Cyclotella (Cyclotella meneghiniana Kutzing, Cyclotella ocellata Pantocsek), Cocconeis (Cocconeis placentula Ehrenberg) and Gomphonema (Gomphonema acuminatum, Gomphonema micropus*)*, and*, matched with the drowning medium used for the experiment. Overall, the number of diatoms recovered from lung tissue samples from group C animals ranged from 3 to 7 frustules/100 μl, while those of groups D and E ranged from 5 to 9 and 10 to 16, respectively (Table 5). The Mann-Whitney test showed no differences in diatoms number between group C and D lung tissue samples. However, the same test demonstrated differences in diatoms number between animals of groups C and E and groups D and E (Table 6), between the experimentally submerged groups and drowned animals and between the control and experimentally submerged groups. In particular, we observed that the diatoms number recovered from lung tissue samples from group E were significantly higher than those observed from animals in groups C and D (*p* < 0.05). Furthermore, we found that the diatom numbers in the lung tissue samples from the experimentally submerged cadavers were significantly lower than those observed in drowned animals and significantly higher than those detected in the control group (Group B) (*p* < 0.05).

**Table 5:** No. of diatoms per 100 μl recovered from animal tissue samples.

**Animals**	**Lung**	**Liver**	**Kidney**	**Brain**	**Spleen**	**Bone morrow**	**Total number of diatoms in organ other than lung**
GC.1^*^	4	0	0	0	0	0	0
GC.2	3	0	0	0	0	0	0
GC.3	4	0	0	0	0	0	0
GC.4	6	0	0	0	0	0	0
GC.5	7	0	0	0	0	0	0
GC.6	3	0	0	0	0	0	0
GD.1	5	0	0	0	0	0	0
GD.2	8	0	0	0	0	0	0
GD.3	5	0	0	0	0	0	0
GD.4	5	0	0	0	0	0	0
GD.5	9	0	0	0	0	0	0
GD.6	5	0	0	0	0	0	0
GE.1	10	0	0	0	0	0	0
GE.2	11	4	0	0	1	0	5
GE.3	12	0	0	0	0	0	0
GE.4	16	0	0	0	0	0	0
GE.5	15	0	0	0	0	0	0
GE.6	15	0	0	0	0	0	0

* *Group C (GC), Group D (GD), Group E (GE)*.

**Table 6:** Mann–Whitney rank sum test of diatom test results in lung.

**Animals**	***P*-value**
Group C vs. D	>0.05
Group C vs. E	<0.05
Group D vs. E	<0.05

## Discussion

The World Health Organization (WHO) defines drowning as a “process of experiencing respiratory impairment from submersion/immersion in liquid” (1). The main mechanism of drowning is rapid and persistent hypoxemia due to the introduction of water or other liquids into the airway (3, 11, 13, 21, 22). Unfortunately, in veterinary literature, only few studies have investigated the macroscopic and microscopic changes following accidental and nonaccidental drowning (3, 23). Overall, the most common injuries observed in drowning cases are located at the respiratory and cardiac level and include pulmonary congestion, pulmonary oedema and pulmonary hemorrhage, foam in the trachea, mouth and nasal cavity, right ventricular distention and drowning medium into the stomach (3, 5, 24–27). Similarly, in our study, the macroscopic and histological examinations of the animals in group A allowed us to observe the same classical injuries of drowning previously described in the human and veterinary literature, such as pulmonary congestion, pulmonary oedema and right ventricular distention (3). Interestingly, similar injuries were also observed in the control and experimentally submerged animals. These findings suggested a low contribution of necropsy and histological techniques in determining the final cause of death in drowning cases. Indeed, as highlighted frequently in human forensic pathology, these injuries, although characteristic of drowning, are not conclusive because they can be detected during autopsies of cadavers dead for causes other than drowning (5). For example, pulmonary oedema or pulmonary hemorrhage as well as right ventricular distention can be observed in a broad range of diseases, such as allergic reactions, neurological diseases, acute kidney diseases or hearth diseases (28–30).

As regard to the diatoms test, in relation to the diatoms recovered from animal tissues, we observed a statistically higher diatom number in the tissues of drowned animals than in the tissue of control animals. In particular, the number of diatoms in drowned animals ranged from 0 to 91 frustules/100 μl, while those in control animals ranged from 0 to 2. Among drowned animals, the highest diatoms number was observed into the organs of the dog dead in the pond located in the countryside region of Venezia. In this case, the diatoms density of the drowning medium was the highest among the assessed drowning mediums. This finding suggested a specific relationship between number of diatoms recovered from water samples and diatoms density in organs tissues samples. It also suggested the possibility of calculating a range of diatoms in the drowning medium at the time of drowning by evaluating the number of diatoms in the victim's organs. However, the small sample size assessed in this study does not allow us to confirm this hypothesis.

In addition, we observed differences between control (Group B) and drowned animals (Group A) regarding diatom location. Indeed, diatoms were detected in the lung, liver and kidney of all animals in group A. Furthermore, diatoms were recovered from spleen of 4 out of 6 animals and from bone morrow tissue samples of 2 animal in group A. Among the assessed organs, the highest diatom number was observed in lung tissue samples, with a range from 27 to 91 frustules/100 μl. In contrast, in animals in group B (control animal), no diatoms were detected in 5 out of 6 cases, while in one case, few diatoms were recovered from liver and spleen tissue samples. Finally, all diatoms detected in the animals in group A matched with the respective drowning medium. The positive diatom results observed in the drowned animals could be explained by antemortem penetration of water containing diatoms into the lungs (3, 10). In these cases, the small size range of diatoms and their morphology allow easy percolation through the alveolo-capillary barrier with subsequent entry into the bloodstream and diffusion to peripherical organs during the preagonic state of drowning (3, 10). In contrast, the positive results observed in control animals could be explained by antemortem diatoms penetrating through the gastro-enteric system as a result of ingestion of diatom-rich food or water (14, 31, 32). Moreover, the inhalation of diatoms should be considered an additional mechanism of diatom penetration in living animals (33, 34). The possibility of false positive diatom results has been previously reported in human forensic pathology (35). However, the human literature on antemortem diatom penetration is highly controversial. Foged et al. (36) in a study conducted on 4 non-drowned human bodies, reported a range of diatoms between 8 and 194 valves/cm in lung, 8 and 54 valves/cm in liver, 7 and 53 valves/cm in kidney, and 15 and 17 valves/cm in femur marrow. However, the most recent studies, conducted with internal protocols to avoid post-mortem diatom contamination, showed results sharply in contrast than those reported by Foged et al. (36). For example, Lunetta et al. (13), in a study conducted on 14 non-drowned bodies, detected only a few diatoms in 6 out of 14 cases. In contrast, Auer and Möttönen (37) reported no diatoms recovered from internal organs of 15 non-drowned cadavers. Similarly, Bortolotti et al. (38) showed the absence of diatoms in both lung and sternum samples of 45 non-drowned cadavers. In contrast, in veterinary forensic pathology, Fucci et al. (10), in a recent study conducted on 10 drowned and non-drowned animals, detected diatoms in otters who died for causes other than drowning. Regarding post-mortem diatom penetration in the experimentally submerged cadavers, diatoms were detected in the lungs of all animals in groups C, D, and E. In addition, diatoms were recovered from liver and spleen tissue samples from 1 animal in group E (72 h in water). All diatoms detected in the in the experimentally submerged cadavers matched with the respective drowning medium. Furthermore, the Mann-Whitney test showed no differences in diatom numbers between lung tissue samples from groups C and D. The same test demonstrated differences in diatom numbers between lung tissue samples from groups C and E and groups D and E. In addition, in all experimentally submerged groups, the number of lung diatoms ranged from 3 to 16 frustules/100 μl and was statistically lower than those observed in drowned animals (group A) and statistically higher than those detected in control animals (group B). Overall, the positive diatom results observed in the lungs of experimentally submerged animals could be explained by post-mortem passive and gravity-dependent movement of water into the lung. In these cases, the absence of cardiac function should prevent diatoms from spreading to other tissues. However, the higher number of diatoms in the lungs of the group E (72 h in water) compared to those of other experimentally submerged groups and the positive diatoms results observed in liver and spleen tissue samples of one animal in group E suggest a specific relationship between post-mortem diatom diffusion and time of permanence in water. Post-mortem changes, such as putrefaction and autolysis, could play an important role in the post-mortem diffusion of diatoms into cadavers. Indeed, diatoms can resist to the putrefaction (12, 39). Therefore, with increasing cadaver decomposition, diatoms could spread into the lungs and enter into the other internal organs of the corpse, miming an antemortem spread due to drowning. Our results are in apparent agreement with those reported by Di Giancamillo et al. (39) that showed the presence of diatoms in heart, lungs, and skin samples of pigs immersed in water for 1 month after death. However, the different assessed species and different times of permanence in water make these findings not directly comparable with the results reported in our study. Overall, the results of this study showed statistical differences between animals in group A and all experimentally submerged groups and control animals regarding diatom numbers recovered from organ tissue samples. Therefore, these findings suggest that the number and location of diatoms may be used as a valid tool to differentiate animals who died in drowning and non-drowning conditions, even if the latter were found in an aquatic environment.

## Conclusion

The diagnosis of drowning is a challenge in human forensic pathology as well as in veterinary forensic pathology. Our results suggest that the diatom test is a valid tool to support the diagnosis of drowning in veterinary forensic pathology. In contrast, the histological and anatomopathological findings in drowning cases are less specific because they can be observed in a wide range of causes other than drowning. Our results showed differences in diatom density and location between drowned and non-drowned cadavers. Therefore, our study provides a reference basis to better establish separation values between drowned and non-drowned animals in veterinary forensic pathology.

## Data Availability Statement

The datasets generated for this study are available on request to the corresponding author.

## Ethics Statement

Ethical review and approval was not required for the animal study because this study was conducted on animals spontaneously dead, so no ethical review and approval was needed.

## Author Contributions

GP drafted the manuscript and contributed to the study concept, study design, analysis and interpretation of data. GP and DD conducted the necropsies and statistical analysis of data. GP and IA conducted the histopathological analysis and diatoms test. RF provided technical and scientific support. OP, DD, IA, FP, AG, NP, RI, and RF revised the manuscript for content and contributed to the interpretation of data. OP also contributed to the study concept and design.

### Conflict of Interest

The authors declare that the research was conducted in the absence of any commercial or financial relationships that could be construed as a potential conflict of interest.

## References

[1] Van Beeck EF Branche CM Szpilman D Modell JH Bierens JJ. A new definition of drowning: towards documentation and prevention of a global public health problem. World Heal Organ. (2005) 83:853–6. 10.1590/S0042-96862005001100015 16302042 PMC2626470

[2] Piette MH De Letter EA. Drowning: still a difficult autopsy diagnosis. Forensic Sci Int. (2006) 163:1–9. 10.1016/j.forsciint.2004.10.027 16378701

[3] McEwen BJ Gerdin J. Veterinary forensic pathology: drowning and bodies recovered from water. Vet Path. (2016) 53:1049–56. 10.1177/0300985815625757 26926081

[4] Lunetta P Modell JH Macroscopical, microscopical, and laboratory findings in drowning victims: a comprehensive review. In: Tsokos M, editor. Forensic Pathology Reviews. New York, NY: Humana Press (2005). p. 3–77. 10.1007/978-1-59259-910-3_1

[5] Farrugia A Ludes B Diagnostic of Drowning in Forensic Medicine, Forensic Medicine - From Old Problems to New Challenges. InTech (2011). Available online at: https://www.intechopen.com/books/forensic-medicine-from-old-problems-tonew-challenges/diagnostic-of-drowning-inforensic-medicine (accessed May 12, 2019).

[6] Bierens JJ Lunetta P Tipton M Warner DS. Physiology of drowning: a review. Physiology (Bethesda). (2016) 31:147–66. 10.1152/physiol.00002.2015 26889019

[7] Shkrum M Ramsay D Forensic Pathology of Trauma: Common Problems for the Pathologist. Totowa, NJ: Humana Press (2007).

[8] Lucci A Campobasso CP Cirnelli A Lorenzini G. A promising microbiological test for the diagnosis of drowning. Forensic Sci Int. (2008) 182:20–6. 10.1016/j.forsciint.2008.09.004 18980820

[9] Byard RW Summersides G. Vitreous humor sodium levels in immersion deaths. J Forensic Sci. (2011) 56:643–4. 10.1111/j.1556-4029.2011.01735.x 21361955

[10] Fucci N Campobasso CP Mastrogiuseppe L Puccinelli C Marcheggiani S Mancini L. Diatoms in drowning cases in forensic veterinary context: a preliminary study. Int J Legal Med. (2017) 131:1573–80. 10.1007/s00414-017-1565-y 28314903

[11] Layon AJ Modell JH. Drowning: update 2009. Anesthesiology. (2009) 110:1390–401. 10.1097/ALN.0b013e3181a4c3b8 19417599

[12] Verma K Role of diatoms in the world of forensic sciences. J Forensic Res. (2013) 4:2–4. 10.4172/2157-7145.1000181

[13] Lunetta P Miettinen A Spilling K Sajantila A. False-positive diatom test: a real challenge? A post-mortem study using standardized protocols. Leg Med (Tokyo). 2013 15:229–34. 10.1016/j.legalmed.2013.03.002 23701706

[14] Yen LY Jayaprakash PT. Prevalence of diatom frustules in non-vegetarian foodstuffs and its implications in interpreting identification of diatom frustules in drowning cases. Forensic Sci Int. (2007) 170:1–7. 10.1016/j.forsciint.2006.08.020 17023133

[15] Anand TP Unmesh AK Diatom test: a reliable tool to assess death by drowning? Int J Res Med Sci. (2016) 4:1479–84. 10.18203/2320-6012.ijrms20161214

[16] Piegari G Iovane V Carletti V Fico R Costagliola A De Biase D Assessment of Google glass for photographic documentation in veterinary forensic pathology: usability study. JMIR Mhealth Uhealth. (2018) 9:e180 10.2196/mhealth.9975 PMC6231880 30249586

[17] Piegari G Prisco F De Biase D Meomartino L Fico R Paciello O. Cardiac laceration following non-penetrating chest trauma in dog and cat. Forensic Sci Int. (2018) 290:e5–8. 10.1016/j.forsciint.2018.07.016 30072043

[18] De Meo S Grassi F Marcheggiani S Puccinelli C Vendetti C Atlante Delle Diatomee Bentoniche nei Corsi D'acqua Italiani. ISPRA, Manuali e Linee Guida 110/2014 (2014). Available online at: http://www.isprambiente.gov.it/it/pubblicazioni/statistiche-download (accessed April 15, 2019).

[19] Stidolph SR Sterrenburg FAS Smith KEL Kraberg A Diatom atlas. In: U.S. Geological Survey Open-File Report 2012–1163 (2012). Available online at: http://pubs.usgs.gov/of/2012/1163/ (accessed April 16, 2019).

[20] Vinyard WC Diatoms of North America. 2nd ed Eureka, CA: Mad River Press (1979).

[21] Conn AW Miyasaka K Katayama M Fujita M Orima H Barker G. A canine study of cold water drowning in fresh versus salt water. Crit Care Med. (1995) 23:2029–37. 10.1097/00003246-199512000-00012 7497726

[22] DiMaio DJ DiMaio VJM Forensic Pathology. Baco Raton, FL: CRC Press (1993).

[23] Banting FG Hall GE Janes JM Leibel B Lougheed DW. Physiological studies in experimental drowning (a preliminary report). Can Med Assoc. (1938) 39:226–8. 20321081 PMC538459

[24] Pollanen MS. Diatoms and homicide. Forensic Sci Int. (1998) 91:29–34. 10.1016/S0379-0738(97)00162-X 9493342

[25] Lindstedt SL Schaeffer PJ. Use of allometry in predicting anatomical and physiological parameters of mammals. Lab Anim. (2002) 36:1–19. 10.1258/0023677021911731 11833526

[26] Szpilman D Bierens JJLM Handley AJ Orlowski JP. Drowning. N Engl J Med. (2012) 366:2102–10. 10.1056/NEJMra1013317 22646632

[27] Munro R Munro HJ. Some challenges in forensic veterinary pathology: a review. Comp Pathol. (2013) 149:57–73. 10.1016/j.jcpa.2012.10.001 23153727

[28] Brown PJ Skuse AM Tappin SW. Pulmonary haemorrhage and fibrillary glomerulonephritis (pulmonary-renal syndrome) in a dog. Vet Rec. (2008) 162:486–8. 10.1136/vr.162.15.486 18408200

[29] Murray JF. Pulmonary edema: pathophysiology and diagnosis. Int J Tuberc Lung Dis. (2011) 15:155–60. 10.5588/ijtld.11.0324-2 21219673

[30] Sureka B Bansal K Arora A Pulmonary edema – cardiogenic or noncardiogenic? J Family Med Prim Care. (2015) 4:290 10.4103/2249-4863.154684 25949989 PMC4408723

[31] Timperman J. Medico-legal problems in death by drowning. Its diagnosis by the diatom method. A study based on investigations carried out in Ghent over a period of 10 years. J Forensic Med. (1968) 16:45–75. 5802631

[32] Hendey NI. The diagnostic value of diatoms in cases of drowning. Med Sci Law. (1973) 13:23–34. 10.1177/002580247301300103 4721834

[33] Spitz WU Schmidt H Fett W. Studies on air filtration strips from different regions of the federal republic for their diatom content. A contribution to the value of diatoms in the diagnosis of death by drowning. Dtsch Z. Gesamte Gerichtl Med. (1965) 56:116–24. 10.1007/BF00577076 14331280

[34] Koseki T Fundamental examinations of experimental materials and control animals on the diagnosis of death from drowning by the diatom method. Acta Med Biol. (1968) 15:207–19.

[35] Shen X Liu Y Xiao C Zheng C Huang J Shi H Analysis of false-positive results of diatom test in the diagnosis of drowning-would not be an impediment. Int J Legal Med. (2019) 133:1819–24. 10.1007/s00414-019-02021-4 30770988

[36] Foged N Diatoms and drowning-once more. Forensic Sci Int. (1983) 2:153–9. 10.1016/0379-0738(83)90104-4 6840638

[37] Auer A Möttönen M. Diatoms and drowning. Z Rechtsmed. (1938) 101:87–98. 3188674 10.1007/BF00200290

[38] Bortolotti F Del Balzo G Calza R Valerio F Tagliaro F. Testing the specificity of the diatom test: search for false-positives. Med Sci Law. (2011) 51:S7–10. 10.1258/msl.2010.010057 22021634

[39] Di Giancamillo A Giudici E Andreola S Porta D Gibelli D Domeneghini C. Immersion of piglet carcasses in water–the applicability of microscopic analysis and limits of diatom testing on an animal model. Leg Med (Tokyo). (2010) 12:13–8. 10.1016/j.legalmed.2009.09.007 19962929

